# Unveiling the anticancer potential of platycodin D

**DOI:** 10.17179/excli2024-7724

**Published:** 2024-10-10

**Authors:** Tae Kyung Hyun

**Affiliations:** 1 Department of Industrial Plant Science and Technology, College of Agriculture, Life and Environment Sciences, Chungbuk National University, Cheongju 28644, Republic of Korea

## ⁯

Triterpene saponins, secondary plant metabolites characterized by a C30 pentacyclic or tetracyclic skeleton, have been extensively utilized across various industries, including medicine, agriculture, and cosmetics, owing to their diverse pharmacological activities. The most common pentacyclic sapogenins have oleanane, ursane, or lupane skeletons, while the use of taraxastane or hopane is considered rare (Podolak et al., 2023[4]). Among oleanane-type pentacyclic triterpene saponins, platycodin D (PD) has potential as a COVID-19 prevention and treatment agent (Kim et al., 2021[2]). Hence, its pharmacological activities are re-evaluated at a heightened level.

PD is one of the main active substances of *Platycodon grandiflorus*, the most commonly used traditional Chinese medicines for lung carbuncle (equivalent to lung abscess nowadays) (Li et al., 2022[3]). The root and aerial parts of *P. grandiflorus* contain PD concentrations of 0.20 % and 0.018 % of dry weight, respectively (Khan et al., 2016[1]). Thus, PD is a critical quality control marker for *P. grandiflorus* roots with Chinese pharmacopeial standards stipulating a minimum required PD content of 0.10 % (Xie et al., 2023[6]). PD has emerged as a promising anti-cancer agent, exhibiting significant cytotoxicity in various human cancer cell lines in both *in vitro* and *in vivo* models (Khan et al., 2016[1]; Li et al., 2022[3]; Xie et al., 2023[6]; Wang et al., 2024[5]). PD exerts its anti-cancer effects through numerous mechanisms, including the inhibition of cellular proliferation and survival pathways, activation of intrinsic and extrinsic apoptotic pathways, promotion of non-apoptotic cell death, cell cycle arrest, autophagy activation, and suppression of angiogenesis and metastasis. Additionally, PD modulates key transcription factors involved in tumorigenesis, underscoring its potential as a multitarget agent in cancer treatment.

This letter provides a concise overview of recent pivotal studies (from 2022 to 2024) exploring the role of PD as a potential anticancer compound (Supplementary Table 1). This summary is anticipated to inspire further research on the development of PD for effective chemoprevention and alternative cancer treatment strategies.

## Conflict of interest

The author declares no conflict of interest.

## Supplementary Material

Supplementary information

## References

[1] Khan M, Maryam A, Zhang H, Mehmood T, Ma T (2016). Killing cancer with platycodin D through multiple mechanisms. J Cell Mol Med.

[2] Kim TY, Jeon S, Jang Y, Gotina L, Won J, Ju YH (2021). Platycodin D, a natural component of Platycodon grandiflorum, prevents both lysosome- and TMPRSS2-driven SARS-CoV-2 infection by hindering membrane fusion. Exp Mol Med.

[3] Li Q, Yang T, Zhao S, Zheng Q, Li Y, Zhang Z (2022). Distribution, biotransformation, pharmacological effects, metabolic mechanism and safety evaluation of platycodin D: a comprehensive review. Curr Drug Metab.

[4] Podolak I, Grabowska K, Sobolewska D, Wróbel-Biedrawa D, Makowska-Wąs J, Galanty A (2023). Saponins as cytotoxic agents: an update (2010–2021). Part II - Triterpene saponins. Phytochem Rev.

[5] Wang R, Wang Y, Liu H, Zhu J, Fang C, Xu W (2024). Platycodon D protects human nasal epithelial cells from pyroptosis through the Nrf2/HO-1/ROS signaling cascade in chronic rhinosinusitis. Chin Med.

[6] Xie L, Zhao YX, Zheng Y, Li XF (2023). The pharmacology and mechanisms of platycodin D, an active triterpenoid saponin from Platycodon grandiflorus. Front Pharmacol.

