# Supplementary material for: Unveiling the anticancer potential of platycodin D
Source: EXCLI J. 2024 Oct 10;23:1232–3. doi: 10.17179/excli2024-7724 (PMC11609939; doi:10.17179/excli2024-7724)
Supplement: Supplementary information [file EXCLI-23-1232-s-001.pdf]

EXCLI Journal 2024;23:1232-1233 – ISSN 1611-2156
Received: August 11, 2024, accepted: September 26, 2024, published: October 10, 2024

Supplementary information to:

Letter to the editor:

UNVEILING THE ANTICANCER POTENTIAL OF PLATYCODIN D

Tae Kyung Hyun

Department of Industrial Plant Science and Technology, College of Agriculture, Life and Environment Sciences, Chungbuk National University, Cheongju 28644, Republic of Korea. Phone: +82-43-261-2520, Fax: +82-43-271- 0413. E-mail: taekyung7708@chungbuk.ac.kr

https://dx.doi.org/10.17179/excli2024-7724

This is an Open Access article distributed under the terms of the Creative Commons Attribution License (http://creativecommons.org/licenses/by/4.0/).

Supplementary Table 1: Recent updates on platycodin D (PD) as a potential anticancer compound

Type of cancer	Key finding	Reference
Colorectal cancer	PD enhanced the sensitivity of KRAS-mutant colorectal cancer cells to cetuximab treatment through the inhibition of the PI3K/Akt signaling pathway, suggesting a potentially reliable theory for improving the efficacy of cetuximab chemotherapy by PD treatment	Liu et al., 2022
	PD decreased LATS2/YAP1 Hippo signaling and survival marker p-AKT expression while increasing the levels of cyclin-dependent kinase inhibitors, such as p21 and p27, ultimately inhibiting proliferation in oxaliplatin-resistant colorectal cancer cells	Wang et al., 2023
	PD inhibited metastasis of KRAS wild-type colorectal cancer cells treated with cetuximab (epidermal growth factor receptor inhibitor) by inhibiting β-catenin, indicating that PD can repress metastasis of colorectal cancer after cetuximab therapy	Lv et al., 2023
	PD decreased the viability of HT-29 colon cancer cells by inducing apoptosis and modulating the MAPK pathway. Specifically, it inhibited ERK and activated p38 and JNK	Han et al., 2024

EXCLI Journal 2024;23:1232-1233 – ISSN 1611-2156
Received: August 11, 2024, accepted: September 26, 2024, published: October 10, 2024

Type of cancer	Key finding	Reference
**Lung cancer**	PD induced the expression of PUMA (p53 up-regulated modulator of apoptosis) through the JNK/AP-1 signaling pathway, thereby promoting apoptosis in non-small cell lung cancer cells	Chen et al., 2022
	PD suppressed angiogenesis and vascular mimicry in non-small cell lung cancer by modulating N7-methylguanosine gene *eIF4E* (eukaryotic translation initiation factor 4E) and associated long non-coding RNAs. Moreover, the combination of PD with ribavirin (an eIF4E inhibitor) exerted synergistic anti-vascular mimicry effects in non-small cell lung cancer by destabilizing the epidermal growth factor receptor	Zheng et al., 2024
**Glioblastoma**	PD induced glioblastoma multiforme cell death by inhibiting autophagy flux, which is involved in the accumulation of low-density lipoprotein (LDL)-derived cholesterol in lysosomes	Lee et al., 2022
	PD inhibited glioma cell proliferation, migration, and invasion by regulating the S-phase kinase-associated protein 2 (Skp2)-p21/p27 signaling axis, which integrates mitogenic and DNA damage signaling to control the entry into the S phase	Li et al., 2023
	PD inhibited the proliferation and motility of glioblastoma cells through the inhibition of the epithelial-to-mesenchymal transition by downregulating DEP domain-containing protein 1B. Hence, PD could be a potential therapeutic option for glioma intervention	Ouyang et al., 2023
**Hematologic malignancies**	PD significantly decreased cell viability in acute myeloid leukemia (AML) cells by triggering mitochondria-dependent apoptosis and G_0/G_1 phase cell cycle arrest through the inhibition of PI3K/AKT and MAPK/ERK signaling pathways. When combined with venetoclax, a B-cell lymphoma 2 inhibitor, PD produced synergistically enhanced cytotoxic effects. The potent anti-leukemic efficacy of PD, which was confirmed using primary samples from *de novo* AML patients, underscores its potential as a promising therapeutic candidate for AML treatment	Jiang et al., 2023
	PD exhibited potent anticancer activity, inhibiting the viability of various diffuse large B-cell lymphoma (DLBCL) cell lines (DB, SUDHL-4, SUDHL-16, Farage, Pfeiffer, OCI-Ly3, OCI-Ly10, and U2932 cells) in a dose-dependent manner by inducing mitochondrial dysfunction and apoptosis, as well as downregulating antiapoptotic proteins. Additionally, PD significantly enhanced the cytotoxicity of venetoclax and markedly suppressed tumor growth in the SUDHL-4-derived xenograft mouse model without observable side effects, offering promising insights for lymphoma therapy	Liu et al., 2023
**Endometrial cancer**	PD effectively reduced the proliferation, invasion, and migration of endometrial cancer cells by upregulating the expression of the α2A-adrenergic receptor (ADRA2A), subsequently inhibiting the PI3K/Akt signaling pathway	Ni et al., 2023
**Breast cancer**	PD administration inhibited the PI3K/Akt signaling pathway by reducing the expression of programmed cell death ligand 1 (PD-L1) on neutrophils, promoting neutrophil apoptosis in mice with 4T1-induced breast cancer. Subsequently, it prevented the establishment of a premetastatic niche and ultimately blocked the development of pulmonary metastasis	Ye et al., 2023

Type of cancer	Key finding	Reference
Gastric cancer	PD suppressed the growth and colony formation capacity of gastric cancer cells by reducing c-Myc protein levels through the activation of the ubiquitin-proteasome degradation pathway	Xu et al., 2023
Prostate cancer	PD induced apoptosis in PC3 human prostate cancer cells by activating both caspase-dependent intrinsic and extrinsic pathways. This effect depended on reactive oxygen species	Choi, 2022
Papillary thyroid carcinoma	PD effectively inhibited the malignant progression of papillary thyroid carcinoma by targeting the NF-κB signaling pathway, thereby enhancing the therapeutic efficacy of pembrolizumab	Deng and Sun, 2022

REFERENCES

Chen S, Wang Q, Ming S, Zheng H, Hua B, Yang HS. Platycodin D induces apoptosis through JNK1/AP-1/PUMA pathway in non-small cell lung cancer cells: A new mechanism for an old compound. Front Pharmacol. 2022;13:1045375.

Choi YH. Induction of ROS-dependent apoptotic cell death by platycodin D in human prostate cancer PC3 cells. J Mens Health. 2022;18:83.

Deng B, Sun M. Platycodin D inhibits the malignant progression of papillary thyroid carcinoma by NF-κB and enhances the therapeutic efficacy of pembrolizumab. Drug Dev Res. 2022;83:708-20.

Han SH, Lee JH, Woo JS, Jung GH, Jung SH, Han EJ, et al. Platycodin D induces apoptosis via regulating MAPK pathway and promotes autophagy in colon cancer cell. Biomed Pharmacother. 2024;172:116216.

Jiang X, Lin Y, Zhao M, Li Y, Ye P, Pei R, et al. Platycodin D induces apoptotic cell death through PI3K/AKT and MAPK/ERK pathways and synergizes with venetoclax in acute myeloid leukemia. Eur J Pharmacol. 2023;956:175957.

Lee SJ, Choi YJ, Kim HI, Moon HE, Paek SH, Kim TY, et al. Platycodin D inhibits autophagy and increases glioblastoma cell death via LDLR upregulation. Mol Oncol. 2022;16:250-68.

Li H, Ouyang J, Liu R. Platycodin D suppresses proliferation, migration, and invasion of human glioblastoma cells through regulation of Skp2. Eur J Pharmacol. 2023;948:175697.

Liu P, Zhao M, Lin Y, Jiang X, Xia T, Li Y, et al. Platycodin D induces proliferation inhibition and mitochondrial apoptosis in diffuse large B-cell lymphoma. Exp Hematol. 2023;123:46-55.e1.

Liu Y, Tian S, Yi B, Feng Z, Chu T, Liu J, et al. Platycodin D sensitizes KRAS-mutant colorectal cancer cells to cetuximab by inhibiting the PI3K/Akt signaling pathway. Front Oncol. 2022;12:1046143.

Lv Y, Wang W, Liu Y, Yi B, Chu T, Feng Z, et al. Platycodin D represses β-catenin to suppress metastasis of cetuximab-treated KRAS wild-type colorectal cancer cells. Clin Exp Metastasis 2023;40:339-56.

Ni Z, Dawa Z, Suolang D, Pingcuo Q, Langga Z, Quzhen P, et al. Platycodin D inhibits the proliferation, invasion and migration of endometrial cancer cells by blocking the PI3K/Akt signaling pathway via ADRA2A upregulation. Oncol Lett. 2023;25:136.

Ouyang J, Li H, Wu G, Hei B, Liu R. Platycodin D inhibits glioblastoma cell proliferation, migration, and invasion by regulating DEPDC1B-mediated epithelial-to-mesenchymal transition. Eur J Pharmacol. 2023; 958:176074.

Wang CH, Baskaran R, Ng SS, Wang TF, Li CC, Ho TJ, et al. Platycodin D confers oxaliplatin Resistance in Colorectal Cancer by activating the LATS2/YAP1 axis of the hippo signaling pathway. J Cancer. 2023;14: 393-402.

Xu Q, Pan G, Wang Z, Wang L, Tang Y, Dong J, et al. Platycodin-D exerts its anti-cancer effect by promoting c-Myc protein ubiquitination and degradation in gastric cancer. Front Pharmacol. 2023;14:1138658.

Ye Y, Xie Y, Pei L, Jiang Z, Wu C, Liu S. Platycodin D induces neutrophil apoptosis by downregulating PD-L1 expression to inhibit breast cancer pulmonary metastasis. Int Immunopharmacol. 2023;115:109733.

Zheng S, Xin Y, Lin J, Xie Z, Cheng K, Wang S, et al. Platycodin D inhibits angiogenic vascular mimicry in NSCLC by regulating the eIF4E-mediated RNA methylome. J Pharm Anal. 2024;14:152-5.
